# Correction: SIRT4 interacts with OPA1 and regulates mitochondrial quality control and mitophagy

**DOI:** 10.18632/aging.101570

**Published:** 2018-09-28

**Authors:** Alexander Lang, Ruchika Anand, Simone Altinoluk-Hambüchen, Hakima Ezzahoini, Anja Stefanski, Afshin Iram, Laura Bergmann, Jennifer Urbach, Philip Böhler, Jan Hänsel, Manuel Franke, Kai Stühler, Jean Krutmann, Jürgen Scheller, Björn Stork, Andreas S. Reichert, Roland P. Piekorz

**Affiliations:** 1 Institut für Biochemie und Molekularbiologie II, Medizinische Fakultät der Heinrich-Heine-Universität, Düsseldorf, Germany; 2 Institut für Biochemie und Molekularbiologie I, Medizinische Fakultät der Heinrich-Heine-Universität, Düsseldorf, Germany; 3 Molecular Proteomics Laboratory (BMFZ), Medizinische Fakultät der Heinrich-Heine-Universität, Düsseldorf, Germany; 4 Institut für Molekulare Medizin I, Medizinische Fakultät der Heinrich-Heine-Universität, Düsseldorf, Germany; 5 IUF - Leibniz Institut für umweltmedizinische Forschung, Medizinische Fakultät der Heinrich-Heine-Universität, Düsseldorf, Germany

Original article: Aging (Albany NY) 2017; 9: 2163-2189

**This article has been corrected:** The authors made a minor mistake in Funding section. Instead of "Deutsche Forschungsgemeinschaft **SFB594** project B09 (to A.S.R)" it should read: “Deutsche Forschungsgemeinschaft **SFB974** B09 (to A.S.R)”

The authors declare that this correction does not change the results or conclusions of this paper. The authors sincerely apologize for this error.

